# Looking at Intergenerational Risk Factors in Schizophrenia Spectrum Disorders: New Frontiers for Early Vulnerability Identification?

**DOI:** 10.3389/fpsyt.2020.566683

**Published:** 2020-10-23

**Authors:** Michele Poletti, Eva Gebhardt, Lorenzo Pelizza, Antonio Preti, Andrea Raballo

**Affiliations:** ^1^Child and Adolescent Neuropsychiatry Unit, Department of Mental Health and Pathological Addiction, Azienda USL-IRCCS di Reggio Emilia, Reggio Emilia, Italy; ^2^Department of Mental Health, ASL Roma 4, Civitavecchia, Italy; ^3^Department of Mental Health and Pathological Addiction, Azienda USL di Parma, Parma, Italy; ^4^Department of Neuroscience, University of Turin, Turin, Italy; ^5^Section of Psychiatry, Clinical Psychology and Rehabilitation, Department of Medicine, University of Perugia, Perugia, Italy; ^6^Center for Translational, Phenomenological and Developmental Psychopathology, Perugia University Hospital, Perugia, Italy

**Keywords:** genetic risk, offspring, serious mental illness, schizophrenia, intergenerational transmission

## Abstract

Offspring of individuals with serious mental illness (SMI) constitute a special population with a higher risk of developing psychiatric disorders, which is also highly prevalent among referrals to child and adolescent mental health services (CAMHS). They often exhibit more or less subclinical conditions of vulnerability, fueled by mutually potentiating combinations of risk factors, such as presumed genetic risk, poor or inadequate affective and cognitive parenting, and low socio-economic status. Despite this evidence, neither specific preventive programs for offspring of parents with SMI are usually implemented in CAMHS, nor dedicated supportive programs for parenting are generally available in adult mental health services (AMHS). Needless to say, while both service systems tend to focus on individual recovery and clinical management (rather than on the whole family system), these blind spots add up to frequent gaps in communication and continuity of care between CAMHS and AMHS. This is particularly problematic in an age-range in which an offspring's vulnerabilities encounter the highest epidemiological peak of incident risk of SMI. This paper offers a clinical-conceptual perspective aimed to disentangle the complex intertwine of intergenerational risk factors that contribute to the risk of developing SMI in offspring, taking schizophrenia spectrum disorders as a paradigmatic example.

## Introduction

Consider the case of M.D., an 11-year-old male offspring of a parent with schizophrenia, whose psychopathological assessment reveals an attenuated psychosis syndrome laying on a schizotaxic subjective substrate of cognitive deficits and pervasive distortions of subjective experiences (aka anomalous self-experiences, ASE) (1, 2). Also, consider the case of C.Z., a 6-year-old female offspring of a parent with schizoaffective disorder, weakly exposed from birth to socially-deprived contexts and taken away from close relatives, that manifests a severe selective mutism when attending kindergarten (from 5 years of age) and subsequently primary school.

In the first case, the psychopathological manifestation of the offspring appears in clear, so-called homotypic, continuity with the psychopathological condition of the parent: i.e., a schizophrenia parent conferring a familiar risk for schizophrenic manifestations, early expressed phenotypically in the offspring in terms of schizotaxia (3), self-disorders (4), and attenuated psychosis syndrome (5). In the second case, i.e., so-called heterotypic continuity, the psychopathological manifestations do not appear in strict continuity across generations (i.e., schizoaffective disorder in the parent, selective mutism in the offspring do not formally align in the same psychopathological cluster), suggesting other pathomorphic factors over and above the putative genetic risk (e.g., environmental factors such as a hypo-stimulating parenting or poor development of social scaffolding).

Starting from these brief vignettes extrapolated from real-world daily clinical practice, it is intuitive how in the mental health domain, the intergenerational transmission of risk is a complex, multi-factorial, and multi-directional phenomenon. Many studies dealt with this phenomenon focusing on specific phenomena such as violence, abuse, child maltreatment, and criminal tendencies (6–10) or on moderating factors as parenting and attachment (11, 12). A more complex issue is represented by the intergenerational transmission of vulnerability for specific psychopathological pictures, characterized by a higher inter and intra categorical heterogeneity in the balance between presumed genetic risk, environment, and their interaction (GxE). In this perspective depression and anxiety have been the most studied conditions (13–16), with most studies focusing on the effects of postpartum depression on parenting and longitudinal offspring mental health.

While common and specific genetic structures of psychopathology have been progressively discovered [e.g., (17, 18)] and unspecific (19–22) and disease-specific (23–26) environmental risk factors (i.e., significant associations) for mental health have been progressively described, pathophysiological mechanisms induced by the interaction between genetic risk and environmental exposure are far from being fully understood and described in humans. Indeed, the complexity of GxE interactions is, for example, represented by the different magnitude scales of putative environmental risk factors, i.e., from micro [such as air pollution and neuroendocrine disruptors: (21, 22)] to macro (such as stress exposure, maltreatment, or parenting). A further layer of complexity resides in epigenetic processes [i.e., the combination of mechanisms that confer short-term and long-term changes in gene expression without altering the DNA code (27)]. These processes become even more critical along neurodevelopment, especially in early years (20), thereby making the intersection between genetic risk and environmental risk factors more complex to decipher and discern. Indeed, epigenetic processes not only mediate the effects of environmental risks in neuropsychiatric disorders but also interact with their genetic load (28–30).

Considering the vastness of the field of intergenerational transmission of risk, this paper offers a clinically digested scoping review focused on schizophrenia spectrum disorders (SSD), a group of severe and chronic mental disorders whose etiology is likely to be multifactorial, with multiple small-effect and fewer large-effect susceptibility genes interacting with environmental risk factors (31).

## Early Vulnerability to Schizophrenia

### Genetics

The earliest approach to genetic risk in schizophrenia has been historically based on the study of offspring, i.e., subjects with first-degree relatives with SSD diagnosis (Familial High Risk): the risk of schizophrenia rises from the 4–6% in second-degree relatives, to 10% in the children of a schizophrenic parent (regardless of whether it is the father or the mother), to 40% in children of both schizophrenic parents up to almost 50% in homozygous twins of schizophrenic subjects (32).

Decades of studies on offspring of individuals diagnosed with schizophrenia robustly showed that they present early endophenotypic alterations detectable at different levels of analysis (e.g., neural, motor, cognitive, emotional, social, and behavioral), placing them in a phenotypical intermediate position between SSD subjects and healthy controls (33–39).

A more recent approach to characterize such genetic risk in quantitative, probabilistic terms is represented by the Polygenic Risk Scores (PRS), i.e., proxy values generated combining multiple genetic markers into a single score indicative of specific lifetime risk for a disease (40); within psychiatry, PRS define cumulative risk profiles based on the identification of genetic variants related to psychiatric disorders, obtained through genome-wide association studies (GWAS) (41, 42). Applied to the general population in developmental years, schizophrenia PRS shows multiple associations with phenotypic expressions through a broad range of soft (i.e., non-psychotic) neurocognitive and behavioral features [for review, (43–46)]. For example, Jansen et al. (47) indicated a selective association between the schizophrenia PRS and higher internalizing tendencies at all ages, as well as with higher externalizing tendency at age 3 and 6; moreover, looking at the syndromic subscales, s-PRS was positively associated with higher emotional reactivity at age 3, with emotional reactivity, anxiety/depression, somatic complaints, withdrawal at the age 6, and with problems of thought at age 10. Another study by Riglin and colleagues (48) reported an association of schizophrenia PRS with performance IQ, speech intelligibility and fluency, and headstrong behavior at age 7–9 years, and with social difficulties and behavior problems at age 4 years.

Overall, studies on schizophrenia PRS suggested that genetic liability is not silent in childhood but is endophenotypically expressed through mild alterations in several domains of functioning. Although intriguing due to the innovative PRS-based approach, these findings substantially replicate and refine those derived from previous familial-high-risk studies on offspring of schizophrenic patients (39), which inspired the original conceptualization proposed by Meehl of schizotaxia, i.e., a broad predisposition to develop SSD due to a genetically predisposed premorbid neurobiological condition (3, 49).

The importance of genetic risk in the neurodevelopmental articulation and clinical unfolding of liability to schizophrenia has been recently reinvigorated by the early detection approach, inspired by the clinical staging model of psychosis (5, 50, 51). Indeed, in addition to attenuated psychotic symptoms and brief limited/intermittent psychotic symptoms, the clinical criteria defining a *Ultra High-Risk* (UHR) of developing psychosis, include a third group, defined by a combination of presumed genetic risk (i.e., family history of psychosis or individual schizotypal personality disorder) associated with a decline in functioning or sustained low decline. This subgroup, termed “*genetic risk and deterioration syndrome*” (GRDS), has a meta-analytical prevalence of 5% among UHR samples (52) and, if not combined with other UHR criteria (i.e., APS or BLIPS), has a relatively lower risk of longitudinal transition to psychosis in comparison with other UHR subgroups.

In sum, the genetic approach to schizophrenia, from earliest studies on offspring to recent PRS studies, globally shows that the presumed genetic load is early expressed in the phenotype but at the same time is not deterministic, conferring a vulnerability that only in the interaction with environmental risk factors may progressively evolve to psychosis and SSD (53), as already classical studies on adopted children pointed out.

### Environment

The Finnish Adoptive Developmental Study (54, 55) followed longitudinally a sample of 185 offspring of schizophrenic mothers who were adopted within the 4th year of life, and compared to a control sample of similar composition in which the adopted children had no biological parents diagnosed with schizophrenia. Findings highlighted an interaction between genetic risk factors and protective factors, with a dimensional distribution of the risk of developing schizophrenia: children raised in adoptive families without severe mental health problems in the adoptive parents, regardless of the genetic risk (presence or absence of schizophrenia in the biological mother), have shown minimum levels of psychopathology over time; the level of psychopathology increased in the presence of an adoptive parent with mental disorders and more in the case of both parents, so much so that of the 35 subjects who had developed longitudinally schizophrenia, 32 had been adopted by problematic and disturbed families.

Similar findings emerged from the Rochester Longitudinal Study (56), which followed up along a 4-year period a group of children of chronically ill schizophrenic women. Mothers varied on mental health dimensions of diagnosis, severity of symptomatology, and chronicity of illness. Other factors included in the analyses were socioeconomic status (SES), race, sex of child, and family size. Curiously, a specific maternal diagnosis of schizophrenia had the least impact on global functioning of children; on the contrary, both SES and severity/chronicity of illness showed a greater impact on development, with children of more severely or chronically ill mothers and lower SES performing most poorly.

The disentanglement of environmental factors impacting on the development of vulnerability to SSD is certainly more complex and nuanced as compared to the investigation of primarily genetic factors. Indeed, as mentioned before, environmental risk factors occur on different scales of magnitude [e.g., from air pollution and neuroendocrine disruptors (19–22) through prenatal/perinatal events as maternal infection and obstetric complications (57–59) to harmful childhood adversities as physical and/or emotional neglect or maltreatment (60, 61) as well as sociodemographic characteristics as urbanicity and immigrant status (62–65)] and are widely dispersed across temporal frames (e.g., from punctiform perinatal events to prolonged exposures in developmental years). Crucially, however, aggregate scores of environmental risk factors (e.g., cannabis use, urbanicity, season of birth, paternal age, obstetric and perinatal complications, childhood adversities), weighted by specific odds ratios for association with psychosis in the literature, may predict transition to psychosis in subjects at familial high genetic risk (66).

Overall, environmental risk factors for SSD are mainly obtained from significant associations in observational studies and their relative weight (odd ratio) may emerge more clearly in meta-analytical studies (24); therefore, especially for environmental risk factors involved in the *developmental origins of health and disease* (DOHaD) hypothesis applied to mental health (20), a necessary step forward to translate scientific insight into tangible preventive strategies is more rigorous experimental designs. Those indeed are essential to properly establish causal inferences and confirm (or disconfirm) the role of putative risk factors (67, 68), such as in the case of the recently confirmed causal role of cannabis use by a mendelian randomization study (69).

With respect to the characterization of combined genetic and environmental risk factors causing the neurodevelopmental pathways leading to increased SSD-proneness, some recent advances are particularly promising. First is the discovery that the schizophrenia PRS score is 5 times greater in those subjects that had experienced perinatal complications, suggesting that a higher genetic risk may increase the likelihood of experiencing prenatal or perinatal adversities (70); second is the preliminary characterization of epigenetic modifications in SSD, as both molecular scars of environmental exposure and source of phenotypic variability (71).

### Modifiable Risk Factors

Within the early intervention paradigm in psychiatry (72), special attention is paid to modifiable risk factors (73, 74), i.e., factors that can be manipulated by early specific and preventive interventions moderating their longitudinal role in contributing to the risk of psychosis and schizophrenia (75). With respect to intergenerational liability, one of the most important modifiable risk factors includes parenting, whose quality is strongly correlated with severity and chronicity of mother mental illness (76, 77), including SSD (78–81). In particular, troubled or high-risk parenting related to serious mental illness may be implicated in increased rates of insecure or disorganized attachment patterns (82–84); these specific attachment patterns, combined with an underlying neurobiological (schizotaxic) vulnerability, may exert a non-protective role for the development of SSD (85–87). However, despite the increasing amount of evidence on the potential role of mental illness on parenting (and therefore on offspring's later risk of mental illness), there is a relative paucity of high-quality studies addressing interventions to reduce the risk of developing mental illness in offspring of parents with mental illness (88). A recent meta-analysis (89) of randomized controlled trials quantified effects of preventive interventions for this at-risk population, reporting small though significant Effect Sizes (ES) for programs enhancing the mother-infant interaction (ES = 0.26) as well as mothers' (ES = 0.33) and children's (ES = 0.31) behavior, that proved to be stable over the 12-month follow-up. Interventions for children/adolescents resulted in significant small effects for global psychopathology (ES = 0.13), as well as internalizing symptoms (ES = 0.17), and increased significantly over time, with externalizing symptoms reaching significance in the follow-up assessments (ES = 0.17). Not surprisingly, interventions addressing parents and children jointly produced overall larger effects than interventions separately focused on offspring or on parents.

## Clinical Translational Implications

As exemplified by the clinical staging model of psychosis (5, 50, 51), severe mental illnesses do not generally arise out of the blue, but rather emerge progressively on the background of complex neurodevelopmental interactions of genetic and environmental factors, often producing early, unspecific premorbid phenotypic alterations, followed along the years by progressively more disturbing prodromal manifestations that finally acquire specific psychopathological and clinical connotations (90). Within this context, children of parents with severe mental illness (such as SSD or mood disorders) represent a peculiar at-risk category, combining presumed genetic liability with an increased likelihood of environmental adversities (from maternal unhealthy lifestyle during pregnancy through prenatal/perinatal complications to exposure to poor parenting).

Modifiable environmental risk factors should be the focus of planned preventive interventions, for example, supporting parents' healthy lifestyle and parenting. These interventions should be integrated within the more general early detection and intervention paradigm, whose focus has gradually become more inclusive, moving from psychosis to trans-diagnostic manifestations of early risk of severe mental illness. Indeed, the prediction of mental illness is moving toward the definition of progressively more accurate risk calculators, combining aggregate scores of multiple risk factors (24, 66, 91). However, as exemplified by the low prevalence of the GRFD subgroup among UHR individuals transitioning to psychosis, the use of genetic liability to establish an a priori risk of mental illness in already help-seeking, putatively prodromal subjects, could be a rather tardive preventive strategy. Instead, the psychopathological risk conferred by such genetic liability should be better deployed to drive psychosocial interventions in those earlier premorbid stages (92, 93), characterized by an increased plasticity and possibility to reduce longitudinal risk.

In this perspective, given the widespread, increasing effort to refine risk phenotypes in order to intervene as soon as possible in a hypothetical primary prevention approach (94), it is paradoxical that, beyond the realm of empirical research, children of parents with mental illness (i.e., a childhood population with an established higher risk for the development of lifetime psychopathology and related risk of biopsychosocial decline) are still only marginally considered in the guidelines and operative policies ruling real-word mental health departments. Indeed, despite this evidence, neither specific preventive programs for offspring of parents with severe mental illness are usually implemented in child/adolescent mental health services, nor dedicated supportive programs for parenting are generally available in adult mental health services. While both service systems tend to focus on individual recovery and clinical management (rather than on the whole family system), these blind spots add up to frequent gaps in communication and continuity of care between these mental health services (95–97). Moreover, the fear of stigmatizing young subjects in relation to possible increased risk for mental illness, although not explicitly acknowledged, characterizes both empirical research and clinical practice (98, 99). Overall, this is reflected in the limited availability of preventive trials and clinical guidelines, as well as in a certain widespread clinical style that—despite formal complacency—widely tolerates a lack of natural or systematic communication between families and clinicians on the stigmatizing issue of mental illness and mental illness risk. For example, clinicians of adult parents will mental illness rarely investigate with the due detail the development and mental health of their offspring; similarly, pediatricians and parents' clinicians seldomly cooperate on the systematic sharing of a comprehensive intervention plan, despite both being aware of the impact of severe and chronic mental illness on families. Notably, despite obvious analogies the attitude is radically different when the parental risk is, for example, linked to a genetic-dependent organic condition (e.g., cardiovascular or oncological diseases). On the contrary, in mental health there is still an ongoing debate on the clinical management of help-seeking subjects at UHR (100, 101), including the opportunity of communicating the risk of psychosis. While respectable, such debate seems to elude an obvious medical fact, that is, although not necessarily transitioning to psychosis, UHR help-seekers usually have a lower level of functioning, poorer quality of life, and a higher proclivity for an array of other mental health disorders. On this background it is crucial to highlight the problem of unmet needs and insufficient managing of early signatures of risk of mental illness in offspring of parents with SMI (102).

In conclusion, a more in-depth (and clinically oriented) appreciation of the intergenerational components building up the predisposition to the development of SMI, exemplified in this perspective paper focusing on SSD, could be an essential step forward toward the next generation of early preventive interventions.

## Author Contributions

The article was jointly conceptualized, wrote, and revised by all authors.

## Conflict of Interest

The authors declare that the research was conducted in the absence of any commercial or financial relationships that could be construed as a potential conflict of interest.
